# Experiences of older people, healthcare providers and caregivers on implementing person-centered care for community-dwelling older people: a systematic review and qualitative meta-synthesis

**DOI:** 10.1186/s12877-023-03915-0

**Published:** 2023-03-31

**Authors:** Lulu Liao, Mingjiao Feng, Yanjie You, Yuqin Chen, Chunyan Guan, Yilan Liu

**Affiliations:** 1 Department of Nursing, Union Hospital, Tongji Medical College, Huazhong University of Science and Technology, Wuhan, Hubei China; 2 School of Nursing, Tongji Medical College, Huazhong University of Science and Technology, Wuhan, Hubei China; 3 Department of Otolaryngology Head and Neck Surgery, Union Hospital, Tongji Medical College, Huazhong University of Science and Technology, Wuhan, Hubei China

**Keywords:** Person-centered care, Older people, Community care, Qualitative meta-synthesis, Systematic review

## Abstract

**Background:**

Person-centered care (PCC) is a critical approach to improving the quality of care for community-dwelling older people. Old-age care services could be provided according to older peoples’ choices, needs, and preferences. The purpose of this study was to synthesize research evidence on the experiences of older people, healthcare providers, and caregivers with PCC and to identify the enablers and barriers to implementing PCC for community-dwelling older people.

**Methods:**

A meta-synthesis of qualitative research design was adopted. Data searches were performed using CINAHL (EBSCOhost), PubMed (OvidSP), Embase (Ovid), Cochrane Database, and PsycINFO (Ovid) in published articles and were reviewed from the earliest date to February 2023. The Qualitative Method Appraisal Tool was used to conduct a quality appraisal on selected articles. Data were extracted based on the capacity, opportunity, and motivation-behavior model (COM-B model), and the findings were synthesized using the meta-aggregative approach.

**Results:**

Twelve included articles were analyzed to identify 122 findings that were organized into 11 categories and combined into three synthesized findings—capacities of older people, healthcare providers, and caregivers; opportunities in the implementation of PCC; motivation in implementing PCC. Capacities consisted of a lack of person-centered knowledge and skills, negative attitudes toward shared decision-making, and a lack of formal training to enhance capabilities among HCPs. Opportunities included a lack of coordination in resource allocation, strengthening multidisciplinary teamwork, establishing a desirable environment, and time constraints. Motivation in implementing PCC included encouraging self-reflection and regulation, respecting the autonomy of older people, lack of clear reward and empowerment mechanisms, and being resilient and optimistic.

**Conclusions:**

The findings of this research provide a reference for implementing successful PCC in the community. The researchers identified barriers and facilitators of implementing PCC, facilitating through stakeholder’s person-centered knowledge and skills being valued and respecting the autonomy of older people. Establishing a positive environment and strengthening multidisciplinary team members also promotes the implementation of PCC. However, additional studies are required to explore the influencing factors and address the barriers.

**Supplementary Information:**

The online version contains supplementary material available at 10.1186/s12877-023-03915-0

## Background

The aged population is rapidly growing worldwide. Approximately 16% of the global population will be over 65 by 2050 [1]. Home and community-based services (HCBSs) embody the core care concept of "aging in place" and combine the humanistic concept using the cost-effectiveness principle [2]. The community-based and person-centered care (PCC) model benefits aged care. PCC is considered a proxy for quality care and has been demonstrated to improve health outcomes [3, 4] and satisfaction of older people [5]. Older people often have multiple care needs with complex health conditions, making them an ideal group to benefit from PCC [6]. Therefore, identifying the enablers and barriers to implementing PCC for community-dwelling older people is crucial.

The term 'PCC' is seen as an umbrella concept that covers the same meaning in this study, such as ‘individualized-centered care’, ‘client-centered care’, ‘resident-focused care’, etc. According to the World Health Organization (WHO), the PCC implies that the individual is viewed as a whole with many needs and goals in caring practice [7]. According to the American Geriatrics Society panel, PCC requires individuals to be motivated to express their values and preferences [8]. Person-centered practice framework of McCormack pointed out “best practices” for PCC included four dimensions: prerequisites (focus on attributes of the care worker); care environment; person-centered processes; outcomes (e.g., satisfaction; feeling of well-being) [9]. Although there is general consensus on the elements of the person-centered, it is necessary to translate the PCC framework into practice [10].

Person-centered care has attracted immense attention in recent years. The WHO has called for person-centered policies to address the complex challenges that individuals face in their communities [11]. A previous systematic review explored the content and essential components of implementing PCC for non-hospitalized older people (65 +) [12], such as treating patients as a whole, shared decision-making, teamwork, and building a PCC foundation. The implementation is also hindered by some factors, such as insufficient educational or institutional help with PCC assessment and care skills [13], resource constraints, less positive attitudes of community doctors [14], limited professional autonomy of HCPs, imbalanced interpersonal contact with older people [15], unprofessional personal qualities of HCPs [4], and the challenge of older people participating in PCC processes [16]. Enablers of PCC implementation include leadership, professional training, organizational support, and appropriate incentives [17]. Furthermore, recent research has shown that family caregivers can benefit from education and support while implementing PCC for people with dementia, which can help those people to be independent [18]. To personalize care, HCPs need to tailor the care plans to meet the needs and preferences of recipients [9]. Due to the complexity of community settings and PCC interventions (involving individuals, organizations, and society), it is difficult to draw accurate conclusions about the influencing factors of implementing PCC in the community based on a single research article.

Previous reviews focused on the concept, elements, key intervention categories, effects of PCC, and whether or not PCC has a relational ethics perspective [4, 19–21]. However, there is no systematic elaboration on the factors influencing the successful implementation of PCC. Qualitative research offers many advantages for an in-depth understanding of the PCC experiences of different stakeholders in the community. Besides, capturing different perspectives of older people, HCPs, and caregivers is more productive for mutual understanding and interactions [22]. The capacity, opportunity, and motivation-behavior model (COM-B model) have been widely used in the medical field to explain and guide various behavioral interventions that can comprehensively and systematically understand the influencing factors in the behavior change process [23]. This study aimed to explore stakeholders' experiences regarding the implementation of PCC in the community and to identify the enablers and barriers based on the COM-B model.

## Methods

### Research design

This systematic review with qualitative meta-synthesis was performed by the guidelines of the Joanna Briggs Institute (JBI) [24]. A meta-aggregative approach to the synthesis of qualitative evidence was used. We used the Procedure PROSPERO (https://www.crd.york.ac.uk/prospero/) to identify published or ongoing projects relevant to the topic. This review was registered with PROSPERO (CRD42022314924). In addition, the reporting of this review was guided by the Enhancing Transparency in Reporting the Synthesis of Qualitative Research (ENTREQ) Statement [25].

### Search strategy

The search strategy aimed to identify peer-reviewed published studies. We conducted a broad bibliographic search using the CINAHL (EBSCOhost), PubMed (OvidSP), Embase (Ovid), Cochrane Database, and PsycINFO (Ovid) from the earliest available date till February 2023. A three-step search strategy was used to locate the literature in this systematic review. First, two researchers undertook an initial limited search of CINAHL and PubMed, followed by a structured analysis of text words contained in titles, and of index terms used to describe the article. With this initial step, we would ensure that our search strategy is sufficiently sensitive, precise, and specific regarding our research objectives as well as the population, concepts, and context of interest. Then, we undertook a structured search using all identified keywords and index terms for a second extensive search. Finally, the reference lists of all included articles were searched manually for additional sources. The initial search included the key search terms of PCC, person-centered care, older people, and community care. Only English language studies were included. Additional file 1 lists the full search strategy.

### Eligibility criteria and study selection

To ensure the correct identification and selection of relevant studies, we developed inclusion/exclusion criteria to aid the selection of relevant papers. The inclusion criteria of the systematic review are as follows: a). all included studies were qualitative or mixed methods studies; b). the context was community home caring organizations providing professional home healthcare visits. Hospitals or nursing homes were excluded; c). the focus was on stakeholders’ experiences participating in PCC intervention programs, including but not limited to older people over the age of 60, HCPs, and family caregivers. These HCPs may have been working in any sector in community health services organizations. Studies only using quantitative methods to analyze data were excluded.

### Quality assessment

Studies were assessed for quality using the JBI qualitative research appraisal tool [26]. Each item could be answered with "yes" (1 point), "no" (0 point), "not applicable" (0 point), or "unclear" (0 point). If the criteria are fully met, the possibility of bias is minimum, which is grade A; if partially meeting the assessment criteria, the possibility of bias is moderate, which is grade B; those which did not meet the assessment criteria at all and had a high possibility of bias is classified as Grade C. To avoid possible biases, lower-quality studies were excluded, similar to previous studies [27, 28]. Original studies had to score more than five assessment criteria to be selected in this review synthesis. Any disagreements were resolved after a discussion between the two reviewers (LLL and GCY) or following consultation with a third reviewer (FMJ) on the team.

### Data extraction and synthesis

All included papers in the systematic review were analyzed independently by two authors. Relevant details for each study were extracted using a standardized data extraction tool from Joanna Briggs Institute Qualitative Assessment and Review Instrument (JBI-QARI). The findings are extracted verbatim from the studies, as are the illustrative quotes usually recorded directly from the participants. Results were graded based on the reader's level of confidence in the findings based on the published data. Two reviewers (LLL and GCY) critically appraised each article independently and attributed a level of credibility to each one. The JBI-QARI qualitative criteria have three levels of credibility: unequivocal (U)—refers to findings that are a matter of fact, beyond a reasonable doubt; credible (C)—refers to findings that are plausible interpretations of the primary data within the theoretical framework; and unsupported (Un)—relates to findings that are unsupported by the data. There were no disagreements between the reviewers in this process. Based on the similarity of meanings, those findings were combined to form different categories; these categories were then subjected to a meta-synthesis to generate comprehensive synthesized findings by meta-aggregation [28]. The first author led a systematic process of data organization and synthesis. After consulting the primary literature, a group discussion to reach an agreement was held in case of a disagreement.

We assessed the final synthesized findings based on the JBI approach for rating the confidence of synthesized qualitative results (ConQual) to determine the confidence level [29]. The summary of findings table was created using the following major elements: population, phenomena of interest, context, synthesized finding, type of research, and the final ConQual scores.

## Results

### Search results

**Fig. 1:**
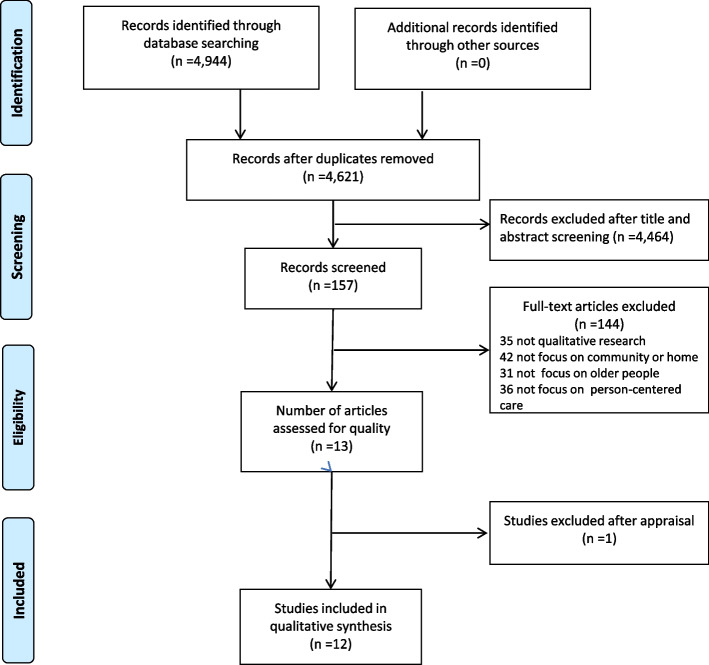
PRISMA flowchart of literature in the search process

We found 4,944 papers after searching relevant databases and grey literature. EndNote X20 software was used to import all search results. After removing 323 duplicates, a total of 4,621 articles were identified. Following the inclusion and exclusion criteria, two trained reviewers independently screened the titles and abstracts, and 4,464 papers were deleted. After a full-text review, 144 of the 157 articles that met the inclusion criteria were eliminated, and 13 met the eligibility criteria. One study was excluded due to quality issues [30]. Figure 1 shows the results of the search and screening strategies. Articles were independently screened by two researchers, and there were no disagreements.

### Characteristics of the studies

Twelve research studies met the eligibility criteria for inclusion in the meta-synthesis, all of which were qualitative studies. The methods applied in the twelve articles were primarily one-to-one interviews and focus group interviews. The articles are spread over time (from 2006 to 2023), mostly concentrated in 2018–2022. Among the twelve papers, four were from Canada, two were from America, two were from Australia, and four were from Ireland, the United Kingdom, the Netherlands, and England, respectively. Table 1 depicts further detailed results of the included studies.

**Table 1:** QARI data extraction of included studies

Author(year)	Methodology	Data generation method(s)	Phenomena of interest	Setting	Geographical	Cultural	Participants	Data analysis
Brown et al., (2006) [31]	Interpretive phenomenology	In-depth interviews	The experiences of nurses on providing a client-centered care	Community living	Ontario, Canada	Canada	8 registered nurses who had in-depth experience in the client-driven care	Hermeneutic analysis
Doody et al., (2013) [32]	Heideggerigan phenomenology	Semi-structured interviews	The registered nurse's care experience in a context where registered nurses provide a client-centered care for older people	Their own homes	Ireland	Ireland	7 participants working in a long-established voluntary service	Thematic analysis
Gillespie et al., (2018) [33]	Interpretive phenomenology	One–one interviews	How can doctors be more caring and how to improve PCC	In two general practice bases (one urban, one rural) in Belfast, UK	Belfast, UK	UK	10 patients (5 female, 5 male)	Template analysis
Uittenbroek et al., (2018) [34]	Qualitative study^*^	In-depth interviews	Experiences of the case managers in implementing PCC programs	GP-practices located in the northern part of the Netherlands	the northern part of the Netherlands	Netherlands	11 case managers	Content analysis
Giosa et al., (2021) [35]	Qualitative study^*^	Semi-structured interviews	Identify how client-centered goal setting practices in home care can be reoriented to older people's self-perceived goals, needs and preferences	Ontario, Canada(The specific location was not reported)	Ontario, Canada	Canada	13 older adults and 12 family/friend caregivers	Thematic analysis
Manalili et al., (2021) [36]	Participatory action research approach	Focus group interviews	Understand the values, preferences and needs of different patients/caregivers for PCC	Community or at the Action Dignity office	Calgary, Alberta, Canada	Canada	66 patients/ caregivers from six communities	Content analysis
McKenzie &Brown (2020) [14]	Qualitative Study^*^	Semi-structured interviews	Identify factors that influence person-centered dementia care for older people with dementia	Office space at the participant's work site or a clinical space	Australia	Australia	12 clinicians (four registered nurses, three occupational therapists and three psychologists, one social worker and one allied health assistant)	Thematic analysis
McKenzie & Brown (2021) [37]	Qualitative Study^*^	Face-to-face interviews	Explore the key skills adopted by clinicians to establish an effective PCC relationship with patients	Workplace or clinical setting close by at the participants convenience	Australia	Australia	12 clinicians (four registered nurses, three occupational therapists and three psychologists, one social worker and one allied health assistant)	Thematic analysis
Hoel et al., (2021) [38]	Phenomenological-hermeneutic approach	Individual in-depth interviews	Experiences with PCC and shared decision-making in older people with dementia	The participant’s own home or at a day-care center	the southeastern part of Norway	Norway	20 older people with moderate to severe dementia	Content analysis
Narayan &Mallinson (2022) [13]	Grounded Theory Study	Face-to-face interviews and online interviews	How home health nurses incorporate PCC principles into their assessment and care-planning practices	General practice base or the online platform	American	American	20 nurses (18 White females,1 Black, 1 male)	Not stated
Stevens et al., (2022) [39]	Qualitative Study^*^	semi-structured interviews	How to provide PCC for care home residents with stroke	The participant’s own home	South London, England	England	28 participants (eight residents with stoke, four relatives of resident, sixteen HCPs)	Thematic analysis
Sareh et al., (2023) [40]	Qualitative Single Case Study	semi-structured interviews	"How" and "why" to apply the PCC approach in the long-term care community	a private community for seniors	Quebec	Canada	8 care providers	Thematic content analysis

^*^ Described as a ‘qualitative study’ if authors could not clearly present a qualitative approach

### Methodological quality

All included studies scored 7–9. The quality appraisal revealed that twelve included articles were rated as B. One of the original studies [30] received only five scores in the quality assessment process due to a lack of adequate details and was therefore excluded from this review (Additional file 2). Table 2 presents the results of the quality assessment.

**Table 2:** Quality assessment of included studies

Citation	Q1^1^	Q2^2^	Q3^3^	Q4^4^	Q5^5^	Q6^6^	Q7^7^	Q8^8^	Q9^9^	Q10^10^	Score	Grade
Brown et al. [31]	U	Y	Y	Y	Y	N	N	Y	Y	Y	7	B
Doody et al. [32]	U	Y	Y	Y	Y	N	N	Y	Y	Y	7	B
Gillespie et al. [33]	Y	Y	Y	Y	Y	N	Y	Y	Y	Y	9	B
Uittenbroek et al. [34]	U	Y	Y	Y	Y	U	N	Y	Y	Y	7	B
Giosa et al. [35]	U	Y	Y	Y	Y	U	N	Y	Y	Y	7	B
Manalili et al. [36]	U	Y	Y	Y	Y	U	Y	Y	Y	Y	8	B
McKenzie and Brown [14]	U	Y	Y	Y	Y	N	Y	Y	Y	Y	8	B
McKenzie and Brown [37]	U	Y	Y	Y	Y	U	U	Y	Y	Y	7	B
Hoel et al. [38]	U	Y	Y	Y	Y	N	Y	Y	Y	Y	8	B
Narayan and Mallinson [13]	U	Y	Y	Y	Y	U	Y	Y	Y	Y	8	B
Stevens et al. [39]	U	Y	Y	Y	Y	N	N	Y	Y	Y	7	B
Sareh et al. [40]	U	Y	Y	Y	Y	N	N	Y	Y	Y	7	B

*Y* Yes, *N* No, *U* Unclear, *N/A* (Not applicable)

^1^ Is there congruity between the stated philosophical perspective and the research methodology?

^2^ Is there congruity between the research methodology and the research question or objectives?

^3^ Is there congruity between the research methodology and the methods used to collect data?

^4^ Is there congruity between the research methodology and the representation and analysis of data?

^5^ Is there congruity between the research methodology and the interpretation of results?

^6^ Is there a statement locating the researcher culturally or theoretically?

^7^ Is the influence of the researcher on the research, and vice- versa, addressed?

^8^ Are participants, and their voices, adequately represented?

^9^ Is the research ethical according to current criteria or, for recent studies, and is there evidence of ethical approval by an appropriate body?

^10^ Do the conclusions drawn in the research report flow from the analysis, or interpretation, of the data?

### Meta-synthesis of qualitative data

**Fig. 2:**
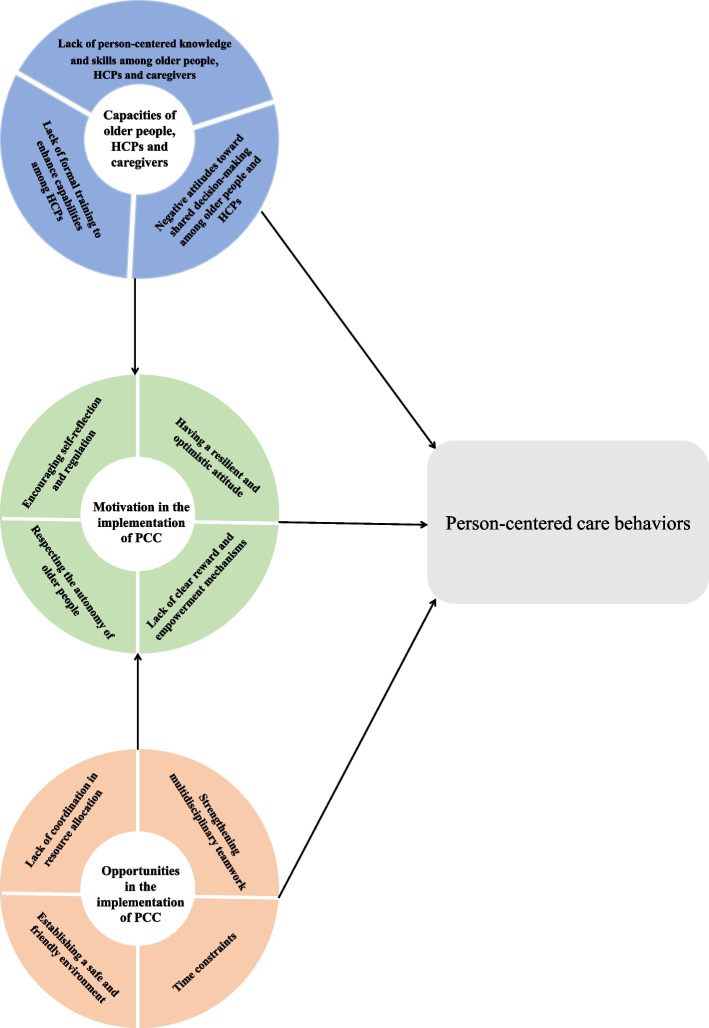
PCC behaviors conceptual map for community-dwelling older people

We extracted a total of 122 findings from the 12 included studies: 106 unequivocal and 16 credible. Those findings were aggregated into 12 categories based on the similarity of meanings, which were then meta-aggregated into three synthesized findings. Figure 2 depicts the final conceptual map. The conceptual map demonstrates that PCC behaviors can be affected in three domains based on the COM-B framework. The capacities of stakeholders and opportunities in implementing PCC can influence the motivation for implementing PCC and as result PCC behaviors. These three elements can also affect the PCC behavior separately. The results obtained from this study are listed in Additional file 3, whereas the results of the meta-synthesis process are shown in Additional file 4. In addition, Table 3 shows the included study from which each theme is derived.

**Table 3:** Themes of Meta-synthesis

Synthesized findings	Category	Brown et al. [31]	Doody et al. [32]	Gillespie et al. [33]	Uittenbroek et al. [34]	Giosa et al. [35]	Manalili et al. [36]	McKenzie and Brown [14]	McKenzie and Brown [37]	Hoel et al. [38]	Narayan and Mallinson [13]	Stevens et al. [39]	Sareh et al. [40]
Capacities of older people, HCPs and caregivers	Lack of person-centered knowledge and skills among older people, HCPs and caregivers			√	√	√			√	√	√	√	√
	Negative attitudes toward shared decision-making among older people and HCPs					√	√			√		√	√
	Lack of formal training to enhance capabilities among HCPs		√								√	√	√
Opportunities in the implementation of PCC	Lack of coordination in resource allocation	√					√			√		√	√
	Strengthening multidisciplinary teamwork	√	√			√	√						
	Establishing a safe and friendly environment	√	√	√	√	√		√		√	√	√	√
	Time constraints		√	√			√				√	√	√
Motivation in the implementation of PCC	Encouraging self-reflection and regulation		√	√	√	√		√					
	Respecting the autonomy of older people	√	√			√	√	√	√	√	√	√	√
	Lack of clear reward and empowerment mechanisms	√			√						√		√
	Having a resilient and optimistic attitude							√	√		√		

*PCC* person-centered care, *HCPs* Healthcare providers

### Synthesized finding 1: Capacities of older people, HCPs and caregivers

It is crucial to recognize that the capacities of older people, HCPs, and caregivers affect the implementation of PCC, including lack of person-centered knowledge and skills, negative attitudes toward shared decision-making, and lack of formal training.

### Lack of person-centered knowledge and skills among older people, HCPs and caregivers (barrier)

Older people, HCPs, and caregivers lack professional knowledge and skills to implement effective PCC. They have an inadequate understanding of PCC and lack communication skills, and implement aged care through their experiences.

*Communication can be number one, no matter who you are looking after.* (McKenzie and Brown, 2021, P.278).

### Negative attitudes toward shared decision-making among older people and HCPs (barrier)

Shared decision-making is the process of older people and HCPs making health decisions together. Some participants stated the HCPs lacked emphasis on shared decision-making. Most HCPs made decisions instead of older people and did not involve older people in developing care plans.

I had a surgery and the doctor that operated on me I met the day of the surgery. They didn't even give me an appointment to meet him or for me to be more informed of the surgery. After the surgery I never saw the surgeon again. (Manalili et al., 2021, P.10).

### Lack of formal training to enhance capabilities among HCPs (barrier)

Meanwhile, some participants stated the community managers should carry out targeted training to improve professional competence among HCPs.

…training increased their knowledge of Major Neurocognitive Disorders, responsive behaviors, and strategies to respond to clients’ unique needs. This information was consistent with the community documents regarding professional development through training and assessing the quality of care. (Zarshenas S et al., 2023, P.8).

### Synthesized finding 2: Opportunities in the implementation of PCC

It is essential to note that opportunities play a significant role in implementing PCC programs. Factors hindering the implementation of PCC include a lack of coordination in resource allocation and time constraints. Furthermore, strengthening a multidisciplinary team facilitates the development of tailored and comprehensive care plans. Establishing a safe and friendly environment can also facilitate the implementation of PCC.

### Lack of coordination in resource allocation (barrier)

Lack of coordination in resource allocation (human and fiscal) can impact the implementation of PCC and, in particular, the choice of intervention measures.

Despite these various resources being available to nurse assistants, they noted that the comprehensive application of resources could be challenging in the short term since these skills are achieved through practice and experience. (Zarshenas S et al., 2023, P.8).

### Strengthening multidisciplinary teamwork (enabler)

Reinforcing multidisciplinary teams and collaboration are an important part of implementing successful PCC program. Older people with diverse diseases often need support in multiple domains, which also facilitates continuity of care for them over time.

One of the greatest barriers is the lack of understanding of person- centeredness. People think that the nurses are the only people to be person-centered but everybody from the maintenance man, cook, household staff and the team all have to be person-centered. (Doody, C et al., 2013, P.1117).

### Establishing a safe and friendly environment (enabler)

Some HCPs stated that establishing a safe living environment is important. Furthermore, the person-centered approach that guided communication might create trust between older people, family caregivers, and HCPs. Significant time spent deliberately building trust and rapport makes sense for implementing PCC programs.

It is important because it’s also a part of building a relationship of trust. Clients apparently like the social aspect, having a nice time. Well, I do think that this is an important component, but it’s certainly not my main reason for coming. (Uittenbroek, R. J et al., 2018, P.6).

### Time constraints (barrier)

Time constraints are opposed to implementing successful PCC programs. This could be partly due to a lack of health staff and bureaucratic overload (lack of a whole service approach, staff turnover, sharp targets, and complex procedures). The situation resulted in high work pressure, overtime, and reduced quality of care.

We’re only touching the tip of the iceberg in relation to person-centeredness. We do our utmost in choice, in documentation, in family involvement, but we would need ten times more staff to do what possibly could be done for each service user to fulfil their dreams, we do the best we can with person centeredness at the core. (Doody, C et al., 2013, P.1117).

### Synthesized finding 3: Motivation in the implementation of PCC

Motivation in PCC programs includes reflexive and automatic motivation [23], which guides one to produce positive or negative emotions toward behavioral goals by increasing knowledge and experiences. Encouragement of self-reflection and regulation in practice leads to self-improvement and provide better care services for older people. Respecting the autonomy of older people and maintaining resilient and positive attitudes contribute to the engagement of all stakeholders in the process of PCC. Furthermore, the lack of clear reward and empowerment mechanisms can reduce staff motivation.

### Encouraging self-reflection and regulation (enabler)

The HCPs can promote self-directed learning through critical thinking skills and engage in self-correction and reflection. Self-reflection is observing and evaluating self-perceptions, emotions, and behaviors [41]. Self-regulation is a motivational mechanism by which an individual's cognitive development moves from a state of disequilibrium to equilibrium [42]. This review refers to how HCPs can reinforce, maintain, or change their behavior based on available rewards or punishments.

I have probably psychoanalyzed a lot of the experiences that I’ve been through to learn from my mistakes. (Narayan MC and Mallinson RK, 2022, P.5).

### Respecting the autonomy of older people (enabler)

Participants reported that HCPs and family caregivers should consider proactive and compassionate care at the heart of their practice and respect the autonomy of older people. They also stated that older people should be treated with respect and dignity while maintaining their autonomy and equality.

Actually, whenever I go to doctor, they call me by name. Once [they] call me by my name I feel close, attached to them. Otherwise, I'm going to feel bad. my relationship with my doctor is really good. (Manalili, K et al., 2021, P.8).

#### Lack of clear reward and empowerment mechanisms (barrier)

Establishing rewards and accountability mechanisms can help foster active HCPs engagement. Some participants stated that the role and task of case managers should be clarified. They should maintain close communication with older people, provide feedback on care plans, and keep an open mind.

Doing the right thing is quality, right thing is a standard. So, if you are diagnosed with particular disease for a patient, then you have to do the right things, what you need to do, so quality, in my opinion he's doing the right things. (Manalili, K et al., 2021, P.10).

#### Having a resilient and optimistic attitude (enabler)

Being resilient and optimistic toward the caring management of older people is a vital step toward activation in PCC programs. The HCPs can overcome the barriers with a resilient attitude that embrace the positive aspects of implementing PCC.

I make it work for me and for the patient. And I can make it work for the agency as well. I’m not afraid to think out of the box, change the way I do things. I am always thinking is there something that I could do differently to be more successful” (Narayan MC and Mallinson RK, 2022, P.6).

## ConQual ‘Summary of Findings’

The ConQual approach was used to assess the confidence level of synthesized findings, including credibility and dependability [27]. The evidence quality of synthesized findings is initially assumed to be high. However, the final confidence for all synthesized findings was low, downgraded by two levels (limitation of included studies). Additional file 5 depicts the ConQual Scores and the summary of the synthesized findings.

## Discussion

This systematic review identified 12 qualitative studies from diverse countries on implementing PCC in the community and included the perspectives of different stakeholders: older people, HCPs, and caregivers. We derived three synthesized findings: capacities of older people, HCPs, and caregivers; opportunities and motivation for implementing PCC. These findings identify the barriers and enablers to implementing effective PCC interventions. This study is timely, considering successful PCC programs might benefit from the quality improvement of care among community-dwelling older people.

### The capacities of older people, HCPs, and caregivers are essential factors affecting the successful implementation of PCC programs

Most participants emphasized the significance of person-centered communication skills and shared decision-making capabilities for implementing effective PCC interventions in the community. Through shared decision-making, older people's values and preferences are combined with the expertise and knowledge of their caring teams [43]. Older people should express their needs and preferences clearly to translate them into professional actions that result in person-centered outcomes [44]. However, it is noteworthy that there is a fine line between negotiating care goals with older people and providing the care they desire [45]. Therefore, appropriate social relationships should be established among older people, family caregivers, and HCPs. Mutual communication must be improved to meet person-centered service imperatives and improve quality care.

The findings of this review also suggest that it would also be useful to provide evidence-based training by combining experience with literature to enhance the professional capabilities of HCPs [46]. The currently available evidence on PCC interventions is not necessarily used in daily clinical practice [47]. Professional training should be designed to integrate theoretical knowledge with practice wisdom. In the process of training, the practicability of theoretical knowledge should be highlighted, trainees' self-thinking and adaptability should be improved [32, 34]. Improving the professional knowledge and skills of HCPs will also, in turn, improve their motivation to participate in PCC programs.

### Opportunities are crucial factors affecting the successful implementation of PCC programs

Most challenges and obstacles exist in the field of opportunity. This review has shown that establishing multidisciplinary teamwork would increase the opportunity to implement individualized care plans. According to the plan-do-check-act (PDCA) cycling management mode, multidisciplinary teamwork and dynamic self-monitoring management are essential for continuous quality improvement [48]. Other studies have also drawn a similar conclusion. For example, Chenoweth and Wu et al. [19, 49] found that collaboration between team members was favorable for fostering a positive environment for meaningful interactions between HCPs and older people. Establishing a safe and friendly environment is an enabler in implementing PCC. The Institute of Medicine (IOM) put forward that "PCC" means that HCPs treat older people as equal partners rather than recipients, establish a trustful and respectful relationship with older people and their families, ensure that older people receive education and support they need, and develop effective care plans [50]. These show the importance of a trusting and welcoming environment for effective PCC implementation.

Time constraint is a barrier to successfully implementing PCC in practice. The essence of time constraints is significantly understaffed and low working efficiency, reflected in lower HCPs to older people ratios and insufficient capacity. The increased health needs and workloads add to the work burden and time pressure. It has been suggested that the shortage of HCPs can be addressed by training more health personnel and by improving working efficiency and quality [51]. Overall, an adequate supply of resources is more conducive to PCC programs; the community infrastructure and positive environment enable older people to experience relative well-being [52].

### Motivation is a necessary component for the successful implementation of PCC programs

Motivation is a mental process that directs behavior [53]. The real action is motivated by the desires of the participants [54], and in the context of implementing PCC interventions, encouraging self-reflection and regulation are crucial for preserving motivation. The present findings suggest that HCPs can learn from their mistakes through self-correction and reflection to create customized interventions more effectively. Ennis showed critical self-reflection, which is the ability to evaluate, analyze and synthesize outside information that can influence our beliefs and actions [55]. Therefore, HCPs can better adapt to changes in unforeseen circumstances by fostering their reflective skills, self-correction, and reflection, which impact effective behavior change regarding PCC interventions. The findings of McKenzie and Brown in this review also affirm the impact and importance of reflective practice on the motivation of HCPs to actively participate in PCC programs [14]. Consequently, it is critical to help HCPs in developing self-reflective skills in PCC interventions.

We found that older people are more willing to cooperate if HCPs respect their autonomy. Clinicians mentioned that older people should exercise their rights, and they should also convey respect and empathy for older people [37]. Jean Watson’s theory of human caring mentioned that each individual should be treated as a whole, their rights respected and treated equally, and the care recipient's self-identity supported [56]. Furthermore, some participants focused on the availability and appropriateness of care for older people. These results are consistent with a previous study that found considerations for access and equity in the healthcare system [57].

Implementing effective PCC interventions for older people is hampered by a lack of clear reward and empowerment mechanisms. The community managers should appropriately authorize HCPs, create a fair, caring, and rule-oriented ethical atmosphere, and establish a firm and reasonable reward-and-punishment mechanism [58]. Empowerment (knowledge, competence, values, impact) and establishment of reward and punishment mechanisms are crucial for promoting the likelihood of sustained lean efforts and improving the desired health outcomes [59].

Maintaining resilience and optimism toward PCC programs was found to increase the motivation of the participants. Some participants expressed concern that HCPs be uncontrollably biased against the patient, which affects the person's perception [14]. Besides, some participants reported that HCPs could adjust their work roles according to their situation and overcome work with a resilient attitude [13]. A previous study also found a resilient attitude capable of adapting to changing situations [60]. Therefore, measures should be taken (e.g., articulating the benefits of PCC and enhancing the practice skills of HCPs) to enhance the positive and resilient attitude of HCPs toward changes.

Qualitative research emphasizes subjectivity and individuality. Meta-synthesis is a systematic review of original qualitative research that can comprehensively interpret the phenomenon. This review provides researchers with the perspectives and experiences of stakeholders regarding PCC interventions as well as an understanding of potential factors that may influence the implementation of PCC programs. However, there are several limitations in this review. First, the included literature is primarily regarded as having moderate dependability because most studies do not provide a statement locating the researcher theoretically or culturally. Second, the number of included studies was too small, and available information may be insufficient. Third, given the limitations of included articles, we did not stratify the analysis by types of HCPs. Fourth, we only included research reported in English, and a potential publication bias may be triggered, which may result in the potential to miss relevant articles. Furthermore, quantitative studies may provide barriers in limitations sections rather than in the results section, so they are also excluded. Finally, the present findings have limited generalizability because the included studies were conducted in developed countries.

## Implications for research

Additional file 6 depicts recommendations from the review, which has been assigned a level of recommendation based on guidelines from the JBI [61]. Three grades of recommendation are used: Grade "A" (strong recommendation), Grade "B" (intermediate recommendation), and Grade "C” (weak recommendation). In order to ensure the success of PCC interventions, stakeholders (HCPs, older people, and caregivers) must have adequate knowledge and competence as well as access to education programs. The community managers should establish and integrate the multidisciplinary team and conduct rational coordination of resource allocation. Therefore, management structures and processes for developing care plans may need to be realigned.

## Conclusion

This study comprehensively synthesized related qualitative evidence that can guide the implementation of PCC intervention programs. A re-conceptualization process was used in the meta-synthesis to understand enablers and barriers in order to provide PCC for community-dwelling older people. The lack of person-centered communication skills, negative attitudes toward shared decision-making, lack of coordination in resource allocation, lack of clear reward and empowerment mechanisms, and time constraints limited the effective implementation of PCC. A supportive environment, positive motivation, and professional educational training would facilitate the implementation of PCC. Considering the above factors as the entry point, community-based interventions could be implemented to improve the practical level of PCC.

## Supplementary Information

**Additional file 1.** 

**Additional file 2.** 

**Additional file 3.** 

**Additional file 4.** 

**Additional file 5.** 

**Additional file 6.**

## Data Availability

The datasets used or analyzed during the current study are available from the corresponding author on reasonable request.

## Abbreviations

HCBSs: Home and community-based services
HCPs: Healthcare providers
PCC: Person-centered Care
WHO: World Health Organization
COM-B model: Capacity, Opportunity, Motivation-Behavior model
JBI: Joanna Briggs Institute
ENTREQ: Enhancing Transparency in Reporting the Synthesis of Qualitative Research guidelines
JBI-QARI: Joanna Briggs Institute Qualitative Assessment and Review Instrument
ConQual: Confidence of synthesized qualitative findings
PDCA: The plan-do-check-act
IOM: The Institute of Medicine
