# Supplementary material for: Experiences of older people, healthcare providers and caregivers on implementing person-centered care for community-dwelling older people: a systematic review and qualitative meta-synthesis
Source: BMC Geriatr. 2023 Mar 31;23:207. doi: 10.1186/s12877-023-03915-0 (PMC10067217; doi:10.1186/s12877-023-03915-0)
Supplement: Supplementary file 1 — Additional file 1. [file 12877_2023_3915_MOESM1_ESM.docx]

**Additional file 1**

Search strategies used for finding qualitative methods articles about implementing person-centered care in community. Number of retrieved articles is given in the right-hand column.

**CINAHL (EbscoHOST)**

| S1 | (MH "Patient-Centered Care") | 5,286 |
| --- | --- | --- |
| S2 | (MH " Client-Centered Care") | 3,710 |
| S3 | (MH" Person-Centered Care ") | 3,766 |
| S4 | AB ((patient* or client* or user) adj2 (participat* or involv* or focused or engag* or interact* or influenc*)) OR ((individuali?ed or personali?ed or patient* or client* or user) N1 (care or nursing or focused)) | 214,791 |
| S5 | S1 OR S2 OR S3 OR S4 | 216,136 |
| S6 | AB (domiciliary N1 (elderly or senior* or geriatric or veteran*)) | 5 |
| S7 | AB (((elderly or senior* or geriatric or veteran*) N3 community) OR ( (elderly or senior* or geriatric or veteran*) N3 home* ) OR ( (elderly or senior* or geriatric or veteran*) N3 house* ) | 9517 |
| S8 | AB (health professionals or healthcare professionals or health personnel or healthcare personnel or nurses or physicians or doctors) | 496,929 |
| S9 | S6 OR S7 OR S8 | 504,812 |
| S10 | (MH "Community Caring") | 1,052 |
| S11 | AB ((home or domicil* or domestic or homecare* or (visiting service*) or community or housebound or in-home or home maker services) and (nursing or care)) | 158,227 |
| S12 | S10 OR S11 | 158,235 |
| S13 | S5 AND S9 AND S12  Limiters‐Clinical Queries: Qualitative‐Best Balance; Language-English | 1,491 |

**Pubmed** (OvidSP)

| 1 | Patient-Centered Care [Mesh Terms] | 23776 |
| --- | --- | --- |
| 2 | Person-Centered Care[Title/Abstract] | 1711 |
| 3 | Client-Centered care [Title/Abstract] | 141 |
| 4 | ((patient* or client* or user or resident or individuali?ed or personali?ed) adj care). ti,ab. | 557 |
| 5 | or/1-4 | 25,553 |
| 6 | home care [MeSH Terms] | 50,540 |
| 7 | community caring [MeSH Terms] | 1,640 |
| 8 | household caring [MeSH Terms] | 336 |
| 9 | domiciliary care [MeSH Terms] | 50,540 |
| 10 | (home or domicil* or domestic or homecare* or community or housebound or in-home) and (nursing or care or service).ti,ab. | 283,076 |
| 11 | or/6-10 | 309,628 |
| 12 | (((elderly or senior* or geriatric or veteran*) and community) or ( (elderly or senior* or geriatric or veteran*) and home* ) or ( (elderly or senior* or geriatric or veteran*) and house* )) .ti,ab. | 11,444,576 |
| 13 | (health professionals or healthcare professionals or health personnel or healthcare personnel or nurses or physicians or doctors).ti,ab. | 626,982 |
| 14 | or/12-13 | 11,786,110 |
| 15 | 5 and 11 and 14 | 4,009 |
| 16 | Qualitative research[Title/Abstract] OR Qualitative study[Title/Abstract] OR ethnography[Title/Abstract] OR phenomenology[Title/Abstract] OR "grounded theory"[Title/Abstract] OR hermeneutic*[Title/Abstract] OR "experience*"[Title/Abstract] OR narrative*[Title/Abstract] OR "action research"[Title/Abstract] OR observation*[Title/Abstract] OR "focus group"[Title/Abstract] OR interview*[Title/Abstract] OR "mixed method"[Title/Abstract] OR multi-method [Title/Abstract] | 2,647,157 |
| 17 | 15 and 16  Filters applied: English | 1,631 |

**Embase（Ovid）**

| 1 | ‘person-centered’/exp | 5,709 |
| --- | --- | --- |
| 2 | ‘patient-centered’/exp | 33,368 |
| 3 | ‘client-centered’/exp | 1,831 |
| 4 | ((patient* or client* or user) N1 (participat* or involv* or focused or engag* or interact* or influenc*)) OR ((individuali?ed or personali?ed or patient* or client* or user) N1 (care or nursing or service)):ab,ti | 5,856 |
| 5 | 1 or 2 or 3 or 4 | 46,188 |
| 6 | ((home or domicil* or domestic or homecare* or (visiting service*) or community or housebound or in-home or home maker services) and (nursing or care)):ab,ti | 92 |
| 7 | 'home health care' OR 'community' | 1,169,707 |
| 8 | 6 or 7 | 1,169,728 |
| 9 | (((elderly or senior* or geriatric or veteran*) and community) or ( (elderly or senior* or geriatric or veteran*) and home* ) or ( (elderly or senior* or geriatric or veteran*) and house* )):ab,ti | 84,621 |
| 10 | 'health professionals'/exp OR 'healthcare professionals'/exp OR 'health personnel'/exp OR 'healthcare personnel'/exp OR 'nurses'/exp OR 'physicians'/exp OR 'doctors'/exp | 2,096,614 |
| 11 | 9 or 10 | 2,164,546 |
| 12 | 'qualitative research' OR 'grounded theory' OR 'observational study' OR 'action research' OR 'ethnography' OR 'phenomenology' OR 'focus group'/exp OR 'interview' OR 'mixed method' OR 'multimethod study' | 868,229 |
| 13 | 5 and 8 and 11 and 12 and [english]/lim | 718 |

**Cochrane Database**

| 1 | [mh ^" Person-Centered Care"] | 940 |
| --- | --- | --- |
| 2 | (centered care or focused care or centered nursing or focused nursing or ((patient* or client* or user) near/2 (participat* or involv* or focused or engag*)) or ((individuali*ed or personali*ed or customi*ed) near/2 care) or ((physician* or nurse* or professional* or doctor* or practitioner*) near/2 (patient* or family or families) near/2 relation*)):ti,ab,kw | 42263 |
| 3 | 1 OR 2 | 42359 |
| 4 | [mh ^" Community Health Nursing"] | 338 |
| 5 | ((home or domicil* or domestic or homecare* or (visiting service*) or community or housebound or in-home or home maker services) and (nursing or care)) :ti,ab,kw | 47164 |
| 6 | 4 OR 5 | 47164 |
| 7 | [mh ^"Aged"] | 242328 |
| 8 | (((elderly or senior* or geriatric or veteran*) and community) or ( (elderly or senior* or geriatric or veteran*) and home* ) or ( (elderly or senior* or geriatric or veteran*) and house* )) :ti,ab,kw | 11657 |
| 9 | (health professionals or healthcare professionals or health personnel or healthcare personnel or nurses or physicians or doctors) :ti,ab,kw | 58919 |
| 10 | 7 OR 8 OR 9 | 299517 |
| 11 | (qualitative OR ethnograph $ OR phenomenology $ OR “grounded theory” OR hermeneutic$ OR “experience$” OR narrative$ OR “action research” OR observation$ OR “focus group$” OR interview$ OR “mixed method” OR “multimethod”) :ti,ab,kw | 150012 |
| 12 | 3 AND 6 AND 10 AND 11  Limits: English | 937 |

**PsycINFO (Ovid)**

| 1 | DE"Person-Centered Care" | 23,608 |
| --- | --- | --- |
| 2 | AB(centered care or focused care or centered nursing or focused nursing or ((patient* or client* or user) near/2 (participat* or involv* or focused or engag*)) or ((individuali*ed or personali*ed or customi*ed) near/2) or ((physician* or nurse* or professional* or doctor* or practitioner*) near/2) | 3,822 |
| 3 | or/1-2 | 6,186 |
| 4 | DE "Home Care Service" | 75,528 |
| 5 | DE "Community" | 22,374 |
| 6 | AB((home or domicil* or domestic or homecare* or (visiting service*) or community or housebound or in-home or home maker services) AND care) | 85,542 |
| 7 | 4 OR 5 OR 6 | 85,836 |
| 8 | AB(((elderly or senior* or geriatric or veteran*) and community) or ( (elderly or senior* or geriatric or veteran*) and home* ) or ( (elderly or senior* or geriatric or veteran*) and house* )) | 24,293 |
| 9 | AB(health professionals or healthcare professionals or health personnel or healthcare personnel or nurses or physicians or doctors) | 202,401 |
| 10 | 8 OR 9 | 223,833 |
| 11 | AB(qualitative OR ethnograph $ OR phenomenology $ OR “grounded theory” OR hermeneutic$ OR “experience$” OR narrative$ OR “action research” OR observation$ OR “focus group$” OR interview$ OR “mixed method” OR “multimethod”) | 922,214 |
| 12 | 3 AND 7 AND 10 AND 11  Limits-Language: English | 167 |

TOTAL FOUND: 4,944
