# Supplementary material for: Experiences of older people, healthcare providers and caregivers on implementing person-centered care for community-dwelling older people: a systematic review and qualitative meta-synthesis
Source: BMC Geriatr. 2023 Mar 31;23:207. doi: 10.1186/s12877-023-03915-0 (PMC10067217; doi:10.1186/s12877-023-03915-0)
Supplement: Supplementary file 2 — Additional file 2. [file 12877_2023_3915_MOESM2_ESM.docx]

**Additional file 2：List of excluded studies**

|  | Study | Reasons for exclusion |
| --- | --- | --- |
| 1 | Higuchi, K. A., Christensen, A., & Terpstra, J. (2002). Challenges in home care practice: a decision-making perspective. Journal of community health nursing, 19(4), 225–236. | Participant's views are rare; research methodology inconsistencies with research objectives and data collection methods; unable to determine where conclusions are drawn from; no evidence of ethics approval and did not present statement locating the researcher culturally or theoretically and the influence of the researcher on the research. |
