# Supplementary material for: Experiences of older people, healthcare providers and caregivers on implementing person-centered care for community-dwelling older people: a systematic review and qualitative meta-synthesis
Source: BMC Geriatr. 2023 Mar 31;23:207. doi: 10.1186/s12877-023-03915-0 (PMC10067217; doi:10.1186/s12877-023-03915-0)
Supplement: Supplementary file 3 — Additional file 3. [file 12877_2023_3915_MOESM3_ESM.docx]

**Additional file 3: Finding and illustrations**

**Brown, D., McWilliam, C., & Ward-Griffin, C. (2006). Client-centred empowering partnering in nursing. Journal of advanced nursing, 53(2), 160–168.**

| Finding | Illustration from study | Evidence |
| --- | --- | --- |
| 1.Caring as the heart of the practice | It is very emotionally draining at times. You know, sometimes you can stay separate from the situation and other times you can’t. You get to know somebody really well and you see them through a difﬁcult time. You feel sadness when someone for whom you’ve played a big role in their life passes away, for instance, or doesn’t do as well as you hoped. (p.163) | Unequivocal |
| 2.Focus on the whole aspect based on client-centredness | You re there with one specific client being more clientcentred, being more focused on what the client is as a whole This lady [with cancer] is palliative so she has cancer. But we are not dealing just with the cancer; we are dealing with her whole life, and her family s reactions. And there are so many other things that we are dealing with rather than just her cancer. (p.163) | Unequivocal |
| 3.On the importance of client’s home environment | Being in somebody’s space, his or her private place, really lets you learn and understand where that client is coming from and why they’re in the place where they are, sometimes, and it just gives you a new insight and it helps you gear your teaching towards that knowledge. (p.163) | Unequivocal |
| 4.On shifting from a providing care expert model to one empowering partnering relationship | We can be with people…it’s different…as opposed to treating the patient, we are being with them. (p.163) | Unequivocal |
| 5.On encountering disempowering on the system level (governmental ﬁnancial constraint and experienced) | The main focus of care, sometimes…is just money-driven. The bottom line is they’re [clients] costing too much, so do something, gets them off the program. Sometimes I think the patient does get lost in the picture. (p.164) | Unequivocal |
| 6.On encountering disempowering on the organizational level (centralized allocation and control of service delivery) | There are a couple of managers who would not allow you to do anything without checking with them…Sometimes you get told more or less what the care plan is. (p.164) | Unequivocal |
| 7.Resources | I have one man who… [telephoned and] said, ‘I dropped my Dosett box’ [which contained 28 prescribed pills per day] …Then I have to call the ofﬁce to get an extra visit to go back and sort out the box. And I just have to struggle to get this visit. (p.164) | Unequivocal |
| 8.Lack of coordination and communication regarding clients' care | We get referrals now from the hospital. We don’t have all the information that we should have…So we just get the person, and they are telling us what [pills they are on]. If they’re a bit confused or if their memory is not great, we don’t know. (p.164) | Unequivocal |
| 9.Unfavourable distribution at individual level (remuneration, workload and working conditions) | One of the very huge issues affecting community nursing right now is ﬁnancial compensation. I mean, you work in a hospital, you do pretty much the same thing you do out here – [but] you get paid more for it  [if you work in the hospital] and it’s a hard thing to convince people that it’s worth coming to the community for personal reward rather than ﬁnancial [remuneration]. (p.164) | Unequivocal |
| 10. Being a bit more understanding for older people | The client has to take responsibility for it [their own care] and I understand all that. It’s just that I think we could be kinder to people [who are frail], patients, clients in the community. I think we could be a bit more understanding. (p.164) | Unequivocal |
| 11.Hegemony of the business model of health services delivery | If you give the clients control within reason, so long as they’re not someone who has a problem [with]…control, they are happy, because they’re getting their needs met. I truly know what their needs are. I know how they want to handle it…I gear my practice to what they need. They’re happy, I’m happy. The case manager is generally satisﬁed unless time and money constraints are saying. Sorry we just can’t do it that way. (p.165) | Unequivocal |
| 12.The orchestrater of resources | The client-driven care, ﬂexible model approach that was brought in…somebody coming in and saying that, you know, ‘Clients can determine what their needs are and when they determine their own needs, they actually use less money and less resources’, and all that sort of thing. I think…certainly the nurse can do it if the client can do it. (p.165) | Unequivocal |
| 13.Delegate authority based on ﬂexible client-driven  care | I sort of examined the way I operate I know I like to be in control. I think that [the need for control] has always been an issue for me. It [control] is a constant thing to let go of and at times, I realize, I need to back off. (p.165) | Unequivocal |

**M Doody, C., Markey, K., & Doody, O. (2013). The experiences of registered intellectual disability nurses caring for the older person with intellectual disability. Journal of clinical nursing, 22(7-8), 1112–1123.**

| Finding | Illustration from study | Evidence |
| --- | --- | --- |
| 14.Individualized care | I always deal with everyone as one person I think that comes with experience. (P.1117) | Unequivocal |
| 15.Individualized service provision and environmental aspects | Coming from big wards with clients all together they did not have personal clothing or personal belongings and may not have had activities. They have now been given the chance in life to have some ownership of their lives. They have their, own rooms, own belongings, own clothes, own routine. They have a say in care- planning and their families are able to visit them in this nice environment but there’re units that don’t have this. (P.1117) | Unequivocal |
| 16. Set goals that the clients can achieve | We set goals during the IPP that the client would be able to achieve, it is invaluable as it brings the people who look after him/her together, the family, MDT, nurses once in the year and the client will be there and part-take. (P.1117) | Unequivocal |
| 17.Limitations to engage in person-centred care (client numbers and lack of a whole service approach) | We’re only touching the tip of the iceberg in relation to person-centeredness. We do our utmost in choice, in documentation, in family involvement, but we would need ten times more staff to do what possibly could be done for each service user to fulﬁl their dreams, we do the best we can with person centeredness at the core. (P.1117) | Unequivocal |
| 18.Organizational teamwork | One of the greatest barriers is the lack of understanding of person- centeredness. People think that the nurses are the only people to be person-centred but everybody from the maintenance man, cook, household staff and the team all have to be person-centred. (P.1117) | Unequivocal |
| 19.Knowing the person | Their health is determined by the staff being observant and knowing them, because if you have new staff, they’re not going to know that the outburst they had today is completely different to what they had a month ago, or the person sitting there doesn’t always sit that way, if you don’t have familiar staff small things slip through the net, they don’t get picked up, be they health issues or otherwise you need familiarity. (P.1117) | Unequivocal |
| 20.On negative impact of staff turnover | I know you have to move, but I think they should consider the elderly person when they’re moving staff because it can be a loss and like a depression in a way, it should be done on a gradual basis and they should always have one or two staff that are solid, that are permanent, that have been with them and knows them inside out, because it makes a difference. (P.1117) | Unequivocal |
| 21.The bio-psycho-social educational model of care from a holistic perspective | You have the spectrum in every area that you go, you get the whole spectrum from the; client, family, emotional, mental health, communication issues whereas when you are working in a general area you’re dealing mainly with physical ailments. (P.1117) | Unequivocal |
| 22.Training and encouraging evidence-based practice | Evidence based practice plays a big role, ﬁrst of all it safeguards the nurse but we are the people that are starting the new practice because we have the older clients, everything has to be evidence-based especially now with the new format for training students and for accountability. (P.1118) | Unequivocal |
| 23.Combine the experiences with education and research literature | It’s a combination of your academic study on that person, your professionalism in getting to know that person. But really and truly it is your day-to-day work, working with that person on a physical, intellectual and intimate level. (P.1118) | Unequivocal |
| 24.More training targeted to the nurses | The number of studies in the area are small, it’s an area that needs looking at and the more it is studied the more use you will get out of it and we have to start selling our uniqueness and stick to it; we know the clients better and we shouldn’t just sit back. Nurse managers have to be more proactive; people get bogged down in the day-to-day work and there’s not enough scope for academia and not enough opportunities given to nurses to do training. (P.1118) | Unequivocal |

**Gillespie, H., Kelly, M., Gormley, G., King, N., Gilliland, D., & Dornan, T. (2018). How can tomorrow's doctors be more caring? A phenomenological investigation. Medical education, 52(10), 1052–1063.**

| Finding | Illustration from study | Evidence |
| --- | --- | --- |
| 25.Assessing the individual | They [doctors] have a better chance of getting it right because they are showing genuine interest in you; you’re not just a number and another box to tick. (Participant D) (P.1059) | Unequivocal |
| 26.Engaged participants by forming relationships | Caring doctors took participants seriously and were not dismissive, unlike doctors whose attitude was uhum, right, sure as they were writing the prescription. Caring doctors were responsive: He interacted with me, at my level. . . he treated me like a sentient, sensible, intelligent woman. . . looking at a situation and him recognising what his responses and reactions needed to be, he was not dismissive, he was engaged. (Participant E) (P.1060) | Unequivocal |
| 27.Time fostered caring | The longer you know someone, the more you care about them. You know, so it is all about getting to know someone. (Participant F) (P.1060) | Unequivocal |
| 28.Constrained caring relationships | General practice has changed, and the chance of you seeing the same doctor every time is remote. (Participant I) (P.1060) | Unequivocal |
| 29.Caring communication skills | They legitimized participants concerns: it was the fact that someone else recognized that. . . at least he knows. . . (Participant G) (P.1060) | Unequivocal |
| 30.Respond to differences between individuals | At the end of the day. . . they are human like  everybody else. You know, we all have good days  and bad days. (Participant D) (P.1060) | Unequivocal |
| 31.Little things that went above and beyond | . . .for a doctor to phone you at tea-time, that’s what struck me. Doctors don’t normally take the time to do that. (Participant A) (P.1061) | Unequivocal |

**Uittenbroek, R. J., van der Mei, S. F., Slotman, K., Reijneveld, S. A., & Wynia, K. (2018). Experiences of case managers in providing person-centered and integrated care based on the Chronic Care Model: A qualitative study on embrace. PloS one, 13(11), e0207109.**

| Finding | Illustration from study | Evidence |
| --- | --- | --- |
| 32.Shift to a person-centered approach | Since I adopted the perspective of the older adults themselves, I’ve been doing things their way, consistent with their own lifestyles and according to their own standards. (DN2) (P.5) | Unequivocal |
| 33.Building a relationship of trust | it is important because it’s also a part of building a relationship of trust. Clients apparently like the social aspect, having a nice time. Well, I do think that this is an important component, but it’s certainly not my main reason for coming. (SW5) (P.6) | Unequivocal |
| 34.Defining the case manager role | You’re right there with the patients and following the processes. You start something up and then you monitor. If it’s not okay, you intervene. (DN1) (P.6) | Unequivocal |
| 35.Maintain a critical overview among case managers | A case manager is someone who maintains a critical overview of everything taking place with regard to the older person. They get things going, and they keep track, checking to see if things have actually been done, and whether they’ve been done properly. They really take a load off others’ shoulders, in my opinion. On the other hand, they are also able to encourage older adults to start doing things themselves. Just helping: “Yes, that’s right,” “You can do it yourself, like this,” and because people often have no idea how things work. And then they can do it themselves. It works both ways: being there for them and encouraging them to start doing things themselves. (DN1) (P.7) | Unequivocal |
| 36.Computer-based individual care and support plan | After a while, I would often decide not to bring the laptop along. Instead, I’d just take a look at the EERS in advance and update it afterwards. I think it interferes with the conversation. If you’re sitting there with a laptop while you’re having a conversation, I think it gets in the way somehow. (DN4) (P.8) | Unequivocal |
| 37.Knowledge and experience | I think the most important thing is to have a good knowledge of the network–knowing where you can turn for things and what the possibilities in your district are. At any rate, so that I (. . .) know where I can find information. If even I don’t know, you can just imagine how difficult it would be for clients. (DN4) (P.8) | Unequivocal |
| 38.Competencies of case managers | (. . .) you’re constantly having to adapt. You have to set aside your own values and beliefs and just observe how others live. This is very important. (. . .) you have to be open, avoid being judgmental. People live their own lives in their own way: Coffee gets reheated; they’ve been doing that their whole life. (. . .) I accept people for who they are; that’s something you have to learn. (DN1) (P.8) | Unequivocal |
| 39.Enhance feedback with colleagues | It’s still in the development stage, and you need feedback. Now and then, you need to be able to talk it out with colleagues to see if you’re on the right track. (DN1) (P.8) | Unequivocal |
| 40.Differences in professional background among older people | I once was visiting a couple and (. . .) they were having trouble filling in a questionnaire. I thought that the questions were too difficult for them, and I just couldn’t understand. And they were having some problems with their house, and they weren’t getting anywhere with them. So, I told [name of the social worker], “I just don’t know what I should do.” [Name of the social worker] went over there for an hour. Neither of these people could read and write  properly, and they were having a real hassle with the housing corporation. No wonder–they couldn’t read the housing corporation’s forms. [Name of the social worker] was able to get this out in the open with her approach and manner of questioning. (DN1) (P.9) | Unequivocal |

**Giosa, J. L., Byrne, K., & Stolee, P. (2021). Person- and family-centred goal-setting for older adults in Canadian home care: A solution-focused approach. Health & social care in the community, 10.1111/hsc.13685. Advance online publication.**

| Finding | Illustration from study | Evidence |
| --- | --- | --- |
| 41.Seeing beyond age enables respect and dignity | And not speak to senior citizens like they're idiots, and don't understand what's going on. Um, being called "dear". Uh … I guess we're just coming into this now, there have been a few ages of things that I run into before, but somebody called me "dear" the other day, and I thought, "Oh, Lord. I have arrived." –Beatrice (older adult) (P.6) | Unequivocal |
| 42.Focused goal-setting | And then they send somebody to check out your home. It- I, I found at the hospital, though, their first, their first … Um, go-to reaction was, "What home are you gonna put him in?" And I'm going, "Oh. Well, I didn't think he was that sick." Like … (laughs) You know, like, uh, it's a stroke, yeah, but his whole right side was, um … what they called 'weakened'. –Karen (caregiver) (P.6) | Unequivocal |
| 43.Relational communication skills involves two-way information sharing | Uh, sometimes they do. We'll- We'll talk and they'll ask me questions about my previous work. I was a reporter and photographer with, uh, the old [name] newspaper office here. I was there for 12 years, and,  I guess, that's where I… No, I always liked books. And, uh, they sometimes ask me about- about my work at the [newspaper office] –Jack (older adult) (P.7) | Unequivocal |
| 44. Share information with colleagues | Oh, God. Wouldn't that be wonderful? A little bio sheet, okay. What did this man do for a living? How many children does he have? Does he have grandchildren? Where were you born? Do you speak two languages? What are your skills? You have hobbies? What religion are you? Are you religious? —Hazel (caregiver) (P.8) | Credible |
| 45.Building trust and understanding | And, uh, it's like, a bit of a bonding like, you know, that, uh, you'll ask them how their children are and, when you've had a blood test or something, they'll say, how did that go or something, you know? –Janice (older adult) (P.8) | Unequivocal |
| 46.Doing ‘with’ instead of doing ‘for’ promotes participation | And I said was there ever, was there any way that I  could have somebody help me with my grocery shopping? And he said 'Oh, yes, you, they have this, uh, you give them a list and they go out and shop and they bring it back and, uh, you pay for it.' But that's not helping with my grocery shopping, that is just grocery shopping. –Doris (older adult) (P.8) | Unequivocal |
| 47.Collaboration is easier when older adults and caregivers lead the way | The priorities here are to just get through a day and put a decent meal on the table once a day and meet his needs because, um, I don't know that they ever stop to think that, you know, "She's here, uh, 21 hours a day with no help. We're only here three hours a day," and, um, you know, if he needs help through the night, guess who's it, you know? I am.–Sue (caregiver) (P.9) | Unequivocal |
| 48.Older adults and caregivers can direct the care environment | Yeah, I know. The one I have, she's a person that really… The warmth affects her a lot. She sweats a lot, so she'll come in the house, and I'm the one that needs it warmer, she'll just open all the windows. And I've told her several times, "It's my house. I will tell you." Now I did have that into her head, (laughs) now she's starting to do it again. –Gail (older adult) (P.9) | Unequivocal |
| 49.Shared decision-making | Uh, I'm diabetic. My heart's a lot better than it was  when I had the heart attack. And, uh, I'm getting close to, uh, I have kidney disease and the doctor is getting me closer to the time where I might have to go on dialysis. I'm- I'm not gonna go on that because I'm 85, uh, I've done everything I've ever wanted to do in my life, and there's no reason why I would want to sit for so many hours and so many days watching my blood go out into a machine and back in. –Jack (older adult) (P.9) | Unequivocal |
| 50.Tailor care activities | We did get a dietitian that was wonderful, that said, "I'm not going to read you the riot act on," said, "What will you eat? Let's work with that." And so, we found things that he will eat, and so they're in his diet. You know. What is it? Yeah. How can we work with you instead of getting you to fit into the system? –Hazel (caregiver) (P.9) | Unequivocal |

**Manalili, K., Siad, F. M., Antonio, M., Lashewicz, B., & Santana, M. J. (2021). Codesigning person-centred quality indicators with diverse communities: A qualitative patient engagement study. Health expectations: an international journal of public participation in health care and health policy, 10.1111/hex.13388. Advance online publication.**

| Finding | Illustration from study | Evidence |
| --- | --- | --- |
| 51.Availability and appropriateness of care | In my mind, uh, we have a pretty darn good health system, the challenge is getting into the system.  (AHS Patient and Family Advisory Member; Group 1, participant 4) (P.8) | Unequivocal |
| 52.Time to access care | The issue was finding a family doctor, there wasn't one available. It was a long wait, but eventually a  friend told me about a doctor and through his referral I had finally found a doctor. (Latino‐Hispanic Community Member; participant 2) (P.8) | Unequivocal |
| 53.‘Free’ healthcare | We have to consider that we are getting this healthcare for free. That's another thing, you know like so many things especially terminal illness many other conditions, so I think we have to keep that in  our mind that you are getting good service in terms of monetary/financial conditions. And its same for everyone…. (South Asian Community Member; participant 5) (P.8) | Unequivocal |
| 54.Insurance, coverage and benefits | She was crying in pain, so from there I had to call an ambulance and I got worried because I didn't have  any money and I was told that ambulance services cost money and that it was expensive… (Latino‐Hispanic Community Member; participant 4) (P.8) | Unequivocal |
| 55.Respectful and compassionate care | Actually, whenever I go to doctor, they call me by name. Once [they] call me by my name I feel close,  attached to them. Otherwise, I'm going to feel bad. my relationship with my doctor is really good. (South Asian Community Member; participant 8) (P.8) | Unequivocal |
| 56.Refusing to share sufficient information with the older people | I asked, like, he asked me questions and he doesn't focus with me. There's no that connection…between you. Like he has to listen to you first, but he's on the computer like “uh huh. What happened to you? — Uh huh”. I don't want that. Like, I want, like, personal connection. He has to understand me. What's my pain…and they don't look. (East African Community Member; participant 7) (P.8) | Unequivocal |
| 57.Patient and caregiver engagement | Yaa, so, being a valued member of the care team, and to be treated as a human being, versus just this patient… cause I had a lot of experience with that. (AHS Patient and Family Advisory Member; Group 1, participant 5) (P.9) | Unequivocal |
| 58.Preferences and expectations for care | Religiously we don't have a problem, but culturally no Somali female wants to face a man. (East African Community Member; participant 4) (P.9) | Unequivocal |
| 59.Equality of care | The system here is really good. Our representatives go to the same hospital as everybody, to the same  hospital we go to. It's a really good thing that the minister will lineup the way we do. (Syrian Community Member; participant 2) (P.9) | Unequivocal |
| 60.Integrated models of care | So, that's one thing I really appreciate, like everything is centralized, you know. I go to any doctor, they just have to open my file and probably see the all history, right. I don't have to keep doing the same test again and again for each and everything. (South Asian Community Member; participant 2) (P.9) | Unequivocal |
| 61.Informed consent | I had a surgery and the doctor that operated on me I met the day of the surgery. They didn't even give  me an appointment to meet him or for me to be more informed of the surgery. I only had further  attention about my condition after the surgery with my family doctor. After the surgery I never  saw the surgeon again. (Latin‐Hispanic Community Member; participant 6) (P.10) | Unequivocal |
| 62.Patient rights | Doing the right thing is quality, right thing is a standard. So, if you are diagnosed with particular disease for a patient, then you have to do the right things, what you need to do, so quality, in my opinion he's doing the right things. (South Asian Community Member; participant 3) (P.10) | Unequivocal |

**McKenzie, E. L., & Brown, P. M. (2020). The provision of person-centred dementia care in the context of mental health co-morbidities: 'It can be upsetting and distressing and it's incredibly sad'. Australasian journal on ageing, 40(2), e133–e142.**

| Finding | Illustration from study | Evidence |
| --- | --- | --- |
| 63.Approach to Care | You're often doing risk assessments in terms of actual physical health and functioning. But then in a more therapeutic sort of side, then I guess with any other client, you'll be trying to build rapport. Participant Seven (Community Team) (P.e136) | Unequivocal |
| 64.Limited control of patient outcomes | In other medical professions it’s a bit like, people die and that’s okay but mental health is like, no, we can’t let people die, that’s the last thing we want to do.  Participant Two (Community Team) (P.e137) | Unequivocal |
| 65.Societal views | I had a lot of questions from people and allied clinicians [asking] ‘what is there for you to do there? What would you possibly be doing there?’ (…) That was really indicative of people’s attitudes.  Participant Twelve (Inpatient Team) (P.e137) | Unequivocal |
| 66.Own biases | Assumptions, biases straightaway. Because I think the moment you hear someone has, you know, we suspect it’s dementia or they’ve got a diagnosis of dementia, straightaway that affects how you perceive the person, whether you want it to or not.  Participant Twelve (Inpatient Team) (P.e138) | Unequivocal |
| 67.Emotional demands | Sometimes I feel like I don’t have anything left to give [my] family at the end of the day if they ring up with a problem, like I really don’t want to know about that. Participant Six (Community Team) (P.e138) | Unequivocal |
| 68.Acceptance | I just get to the point some days and I think we’ve done as much as we can and we can’t do anymore.  Participant Eleven (Inpatient Team) (P.e139) | Unequivocal |
| 69.Reflective practice | I think we’re very lucky here, we’ve got a very good team I like to debrief about things later (…) I’ll just have a chat to one of the other clinicians or the case manager. Luckily this team’s very open to that.  Participant Three (Community Team) (P.e139) | Unequivocal |
| 70. Separate your work environment from your home | I have found in the past, going to the gym on the way home from work is good because it kind of separates work and home, you're doing something in the - in between or putting music on in the car or practising mindfulness. Participant Six (Community Team) (P.e139) | Unequivocal |

**McKenzie, E. L., & Brown, P. M. (2021).** **"Just see the person who is still a person (…) they still have feelings": Qualitative description of the skills required to establish therapeutic alliance with patients with a diagnosis of dementia. International journal of mental health nursing, 30(1), 274–285.**

| Finding | Illustration from study | Evidence |
| --- | --- | --- |
| 71.Empathy | Whether they can talk or they can’t talk, they can trust you, they feel safe and secure. That’s the vital thing, being safe and secure and feeling it. (P.278) | Unequivocal |
| 72.Unconditional positive regard | And then just if she’s not, the idea will be whether we can have the conversation today or whether it’s the next time. It’s a non-judgmental conversation. It’s like, what got in the way, what was it about that morning? Were you really anxious, did you have a terrible night’s sleep? Do you think it was a good idea at the time to go for coffee but now you can’t face it? If you can’t face it why can’t you face it? (P.278) | Unequivocal |
| 73.Congruence | The most important part of treatment is your therapeutic relationship. I understand that you’ve got to keep that, you can’t go home and like, cry about everybody; you wouldn’t be able to do your job. But I think - and I know, the most important things is authentic care. (P.278) | Unequivocal |
| 74.Psychological ﬂexibility | I’m still learning but I think it is more of an acceptance (. . .) I’m going to have to just do things intuitively and just relax a bit about not going in with my eight weeks thing and trying to force that on people. So, it’s a strategy of acceptance for me that I need to well, I need to go with the ﬂow a little bit. (P.278) | Unequivocal |
| 75.Communication skills | Communication can be number one no matter who you’re looking after. (P.278) | Unequivocal |

**Narayan MC, Mallinson RK: Home Health Nurses' Journey Toward Culture-Sensitive/Patient-Centered Skills: A Grounded Theory Study. HOME HEALTH CARE MANAGEMENT AND PRACTICE 2022, 34(1):24-34.**

| Finding | Illustration from study | Evidence |
| --- | --- | --- |
| 76.Caring values | “You have to be a people-person and you have to come to patients from a curious perspective, not a judgmental one” [P14]. (P.5) | Unequivocal |
| 77.Caring relationship | I build trust by really listening to what they’re trying to tell me because that unravels so many things about why they are, or are not, doing something” [P7] (P.5) | Unequivocal |
| 78.Enhance culture competency | I grew up an Army brat and my mom’s German. And a lot of my mom’s friends are Asian. I was exposed to a lot of cultures that other children weren’t. I think that’s helped me with my cultural competency” [P10]. (P.5) | Credible |
| 79.Being sensitive to the needs of different people | [I learned] to be culturally sensitive by being a homecare nurse. You see so many different people that you just roll with it. You get used to it. You take it in” [P3]. (P.5) | Unequivocal |
| 80.Creative/critical reflection | I have probably psychoanalyzed a lot of the experiences that I’ve been through to learn from my mistakes” [P11]. (P.5) | Unequivocal |
| 81.Inadequate education in nursing school | I remember being in nursing school and we had a  very, very, very short module about cultural diversity and it did not do what it should have done. It was so small, but we live in a world where the cultural diversity is so large [P18]. (P.6) | Unequivocal |
| 82.Develop skills through good mentors | I think that as a home health nurse – working independently – it is hard to develop [home health CS/PC] skills. We need good mentors [P10]. (P.6) | Unequivocal |
| 83.Agency support for patient-centered care | I feel as though the mission statement really resonates with nurses. Before you do anything, you should be following our mission, our values [P11]. (P.6) | Unequivocal |
| 84.Lack of time | I think in home care today, everybody is overworked, overwhelmed. One cannot work like that and give proper care. I hear continuously, ‘Too many patients, not enough time’ [P17] (P.6) | Unequivocal |
| 85.Productivity and compensation | The focus is on productivity. The focus is on ‘get as many patients seen as possible.’ It pushes people to go faster and that makes it harder to give patient-centered care, because you’re rush rush rush rush rush. You’re like, ‘I’ve got to get into this one, then I’ve got to go see this one. ‘Boom boom boom boom boom’[P5] (P.6) | Unequivocal |
| 86.Documentation systems and requirements | It’s incredibly burdensome and the time that it takes to chart overwhelms the time that you have with the patient [P20]. (P.6) | Unequivocal |
| 87.Business orientation to patient care | The business model they impose on us doesn’t jive with patient-centered care [P3] (P.6) | Unequivocal |
| 88.Side-stepping the barriers with a resilient attitude | A couple of nurses, who worked for agencies with preceptorship programs, sidestepped aggressive productivity requirements, by working as preceptors or parttime educators [P11, P13]. (P.6) | Unequivocal |
| 89.Transcending barriers with a resilient attitude | I make it work for me and for the patient. And I can make it work for the agency as well. I’m not afraid to think out of the box, change the way I do things. I am always thinking is there something that I could do differently to be more successful” [P13] (P.6) | Unequivocal |

**Hoel KA, Rokstad AMM, Feiring IH, Lichtwarck B, Selbæk G, Bergh S. Person-centered dementia care in home care services - highly recommended but still challenging to obtain: a qualitative interview study. BMC Health Serv Res. 2021 Jul 22;21(1):723. doi: 10.1186/s12913-021-06722-8. Erratum in: BMC Health Serv Res. 2022 Jul 22;22(1):939. PMID: 34294078; PMCID: PMC8299610.**

| Finding | Illustration from study | Evidence |
| --- | --- | --- |
| 90. Inadequate comprehension of PCC in the home care services programs | I do not know their (the home care service’s) plan, what it is, whether they have scheduled days for each one, or whether it is only occasionally that they are coming. I do not know. But at least I want them to come a little more often. (11) (P.5) | Unequivocal |
| 91. Value on the visits of the homecare providers | They come by, talk and then they go again, cheerful and nice. (4). (P.5) | Unequivocal |
| 92. the staff were nearby and being safe | They are coming in the morning and in the evening,  I appreciate that. I feel taken care of ... And that's very reassuring.” (08) (P.5) | Unequivocal |
| 93. The importance of the quality of the relationship with the staff | If there are new ones (staff), you have to break the ice. This is not necessary if we know each other” (10). (P.5) | Unequivocal |
| 94. The desire to live in their own home seemed to increase their effort to adapt to the home care service | I'm very grateful, I am, because you never know …  And in this case, in my situation, you cannot predict anything. You must take the day as it comes and live through it until the next day. But I am very happy that the home care service is coming.” (11) (P.5) | Unequivocal |
| 95. Not be a nuisance to the staff by asking for extra time and help | I'm waiting; it's not just me, you know, so it might be a little late at night before they are available. Then they are tired and are about to go home. I understand that.” (11) (P.6) | Unequivocal |
| 96. The care that they received was predetermined and determined independent of the participants’ wishes and needs | They usually come and have their routines, some to wash and all that stuff. Beyond that, I do not know.  These ladies who come and want to talk, so I cannot exactly say anything about it … but I manage most things on my own.” (09) (P.6) | Unequivocal |
| 97. The older people were not sure about how much they could participate in decisions related to the service | “I think they have mostly decided in advance the service I get. Maybe I can be involved in making decisions, but I have not tried.” (06) (P.6) | Unequivocal |
| 98. The service had limited opportunities for involvement and individualized tailored services | The final interpretation of the findings identified that the participants appreciated the possibility to stay safely in their own homes, and most experienced good support from staff with few unmet needs. However, they expressed various views and understandings of the service and had limited opportunities for involvement and individualized tailored services. | Credible |
| 99. It is difficult for people with dementia to understand and influence PCC | The overall theme identified in summarizing the findings was: “It is difficult for people with dementia to understand and influence home care services, but the services facilitate the possibility to stay at home and feel safe with support from staff.” | Credible |

**Stevens E, Clarke SG, Harrington J, Manthorpe J, Martin FC, Sackley C, McKevitt C, Marshall IJ, Wyatt D, Wolfe C. The provision of person-centred care for care home residents with stroke: An ethnographic study. Health Soc Care Community. 2022 Nov;30(6):e5186-e5195. doi: 10.1111/hsc.13936. Epub 2022 Jul 23. PMID: 35869786.**

| Finding | Illustration from study | Evidence |
| --- | --- | --- |
| 100. Lack of formal training | A manager explained how staff might receive ‘indirect training’ (Man02) from clinical specialists via their input into care plans.(e5189) | Credible |
| 101. Lack of professional knowledge | In general, they were unaware of other common long-term consequences of stroke, including cognitive and visual impairment and emotional/psychological problems. (e5189) | Credible |
| 102. Lack of sensitivity to the specific needs of older people | [staff would say to the general practitioner, GP] ‘[residents with stroke] has swallowing difficulties and you've given him all tablets so could you change it to liquids’ […] that's what person-centered care is all about. (Man02) (e5190) | Unequivocal |
| 103. The importance of residents being permitted or enabled to do things for themselves | We try to let them be as independent as they possibly can for as long as they can (SCW02). (e5190) | Unequivocal |
| 104. Planned activities were not differentiated by ability or inclusive | …it's all right for everybody else that hasn't had a stroke, I can't do what other people are doing. (Res08) (e5191) | Unequivocal |
| 105. Respect the autonomy of older people | Music is being played in the lounge. [Res06] stands up with a hand on their walking aid. A care worker comes over. She begins dancing, taking hold of [resident]’s hand to prompt them to join in, which they do with a smile. [Res06] tells her ‘You are a sister to me now, not a friend’. (Observation excerpt, Res06) (e5191) | Unequivocal |
| 106. Residents' interests and preferences were not always supported | Overall, activity in the homes revolved around care routines and staff availability. One resident (Res01) (a wheelchair user) complained they had no opportunity to do any gardening tasks in the home's garden. (e5191) | Credible |
| 107. Lack of interactions with residents with  stroke-related communication impairment | One nurse also observed this and commented that staff needed to allow more time for residents with aphasia to communicate: We're asking and we're answering now ourselves for them and they're supposed to just with their eyes say I agree with what you say’ […] after stroke people, they have [aphasia] […] they try and it's not coming out and [staff say] ‘what you say? What you say?’ and they [resident] stop talking. (Nu01)(e5191) | Unequivocal |
| 108. Time pressures on care | …the physio will come and make a plan, like every day ten minutes do this exercise. Never ever seen [staff] stay and do [the exercise] […] They'll say ‘I'm busy’. (Nu02) (e5192) | Unequivocal |
| 109. Staff shortages increased time pressure | [SNu01] is seated behind the desk filling in medication forms. They are angry. [SNu01] tells me they were the only [staff member] on the ground floor today – that is why they have to retrospectively fill in the medication. The two [care workers] they were meant to have had not turned up.  (Observation excerpt, SNu01) (e5192) | Unequivocal |
| 110. Funding was a key limitation to delivering better care | The [care worker] can't interact with everyone and she has so much things to do, paperwork, training, caring. […] If someone really wants to deliver the best care, I think you need to look into getting more funding to activity. (AC01) (e5192) | Unequivocal |

**Zarshenas S, Paulino C, Sénéchal I, Décary J, Dufresne A, Bourbonnais A, Aquin C, Bruneau MA, Champoux N, Belchior P, Couture M, Bier N. Application of the Person-Centered Care to Manage Responsive Behaviors in Clients with Major Neurocognitive Disorders: A Qualitative Single Case Study. Clin Gerontol. 2023 Jan 2:1-13. doi: 10.1080/07317115.2022.2162468. Epub ahead of print. PMID: 36591952.**

| Finding | Illustration from study | Evidence |
| --- | --- | --- |
| 111. A homelike atmosphere | This community has private bedrooms, a kitchen, a conference room for social gatherings, a living room, adapted bathrooms with grab bars and transfer bath benches, and a chapel. Its open-concept interior design, with the kitchen and living room at the center and bedrooms surrounding them, prevents clients from becoming lost in hallways, while encouraging socialization. NAs explained that having the kitchen at the center of the building encourages clients to sit around kitchen tables and communicate with each other, which may also create a sense of unity.(p.4) | Credible |
| 112. A safe environment | For instance, they created a clutter-free outdoor environment, and increased its privacy with a cedar hedge. Furthermore, all emergency equipment is available inside and outside of the building, while main entrances are supplied with ramps and grab bars to facilitate the use of mobility aids (e.g., walkers, wheelchairs, and crutches).(p.5) | Credible |
| 113. Encouraging effective communication | Participants mentioned that showing empathetic behavior and respect for clients’ dignity may help shape better communication and strengthen relationships with them.“I am a very empathetic person. . I put myself in the patient's shoes and I do what I would want done for my grandmother, for my mother and for myself later on.”  (NA 4).(p.6) | Unequivocal |
| 114. Providing different options to facilitate older people involvement | This statement was confirmed during observation of NAs, when they encouraged clients to participate in daily living activities (e.g., setting the table at mealtime). (p.7) | Credible |
| 115. Tailoring activities to the clients’ needs and values | There is a lady who has difficulty waking up in the morning. [...] I open her room curtain, I pull back her sheets a little bit, and greet her by saying “hello my friend”! [...]. She's comfortable into her covers. I'll take the time to chat with her.” (NA1)(p.7) | Unequivocal |
| 116. Providing them with different levels of assistance by taking into account their disability levels and comorbidities | To support clients to be independent, NAs provide  them with opportunities to be involved in simple  tasks such as closing dress buttons under the care providers’ supervision. Also, clients are invited to pick up their plate, and when possible, they are allowed to choose the side dish or food dressings such as condiments for their burgers. (p.8) | Credible |
| 117. Empowering care providers | Making various resources available to NAs was considered imperative to facilitate the effective application of the PCC approach within the LTC community. (p.8) | Credible |
| 118. Flexibility of time spent with clients | They can adjust activities based on clients’ pace to decrease potential RBs (e.g., during bathing). They will have enough time to shape the trusted bond, customize interventions based on clients’ needs, and involve them in activities.(p.8) | Credible |
| 119. Heavy workload and low caseload | NAs mentioned that various factors, such as a low caseload, might contribute to expanding this flexibility. Also, similar client functional profiles may allow a balance between the time invested in assisting them with their daily living activities and other activities. However, the fact that NAs are responsible for performing housekeeping duties in addition to their professional roles was identified as an obstacle that may prevent them from spending enough therapeutic time with clients.(p.8) | Credible |
| 120. Equipment and training | NAs reported that training increased their knowledge of MNDs, RBs, and strategies to respond to clients’ unique needs. This information was consistent with the LTC community documents regarding professional development through training and assessing the quality of care. (p.8) | Credible |
| 121. A desirable work environment | NA1 mentioned, “Although the salary is not high, I wouldn't change my workplace for all the money in the world. There's a nice quality of life here.(p.8) | Unequivocal |
| 122. The importance of resources being available | Despite these various resources being available to  NAs, they noted that the comprehensive application of resources could be challenging in the short term since these skills are achieved through practice and experience.(p.8) | Credible |
