# Supplementary material for: Experiences of older people, healthcare providers and caregivers on implementing person-centered care for community-dwelling older people: a systematic review and qualitative meta-synthesis
Source: BMC Geriatr. 2023 Mar 31;23:207. doi: 10.1186/s12877-023-03915-0 (PMC10067217; doi:10.1186/s12877-023-03915-0)
Supplement: Supplementary file 4 — Additional file 4. [file 12877_2023_3915_MOESM4_ESM.docx]

**Additional file 4: Results of meta-synthesis**

| **Finding** | **Category** | **Synthesized findings** |
| --- | --- | --- |
| 29.Caring communication skills (U) | Lack of person-centered knowledge and skills among older people, HCPs and caregivers | Synthesized findings 1: Capacities of older people, HCPs and caregivers  It is crucial to recognize that the capacities of older people, HCPs and caregivers affect the implementation of PCC, including lack of person-centered knowledge and skills, negative attitudes toward shared decision-making and lack of formal training. |
| 32.Shift to a person-centered approach(U) |  |  |
| 37.Knowledge and experience(U) |  |  |
| 38.Competencies of case managers(U) |  |  |
| 40.Differences in professional background among older people(U) |  |  |
| 43.Relational communication skills involves two-way information sharing(U) |  |  |
| 75.Communication skills(U) |  |  |
| 90.Inadequate comprehension of PCC in the home care services programs(U) |  |  |
| 99.It is difficult for people with dementia to understand and influence PCC(C) |  |  |
| 101. Lack of professional knowledge(C) |  |  |
| 113. Encouraging effective communication (U) |  |  |
| 49.Shared decision-making(U) | Negative attitudes toward shared decision-making among older people and HCPs |  |
| 50.Tailor care activities(U) |  |  |
| 56.Refusing to share sufficient information with the older people (U) |  |  |
| 57.Patient and caregiver engagement(U) |  |  |
| 96.The care that they received was predetermined and determined independent of the participants’ wishes and needs(U) |  |  |
| 97. The older people were not sure about how much they could participate in decisions related to the service(U) |  |  |
| 103.The importance of residents being permitted or enabled to do things for themselves(U) |  |  |
| 114.Providing different options to facilitate older people involvement(C) |  |  |
| 21.The bio-psycho-social educational model of care from a holistic perspective(U) | Lack of formal training to enhance capabilities among HCPs |  |
| 22.Training and encouraging evidence-based practice (U) |  |  |
| 23.Combine the experiences with education and research literature（U） |  |  |
| 24.More training targeted to the nurses(U) |  |  |
| 78. Enhance culture competency (C) |  |  |
| 81.Inadequate education in nursing school(U) |  |  |
| 82.Develop skills through good mentors(U) |  |  |
| 100. Lack of formal training(C) |  |  |
| 120. Equipment and training(C) |  |  |
| 7.Resources(U) | Lack of coordination in resource allocation | Synthesized findings 2: Opportunities in the implementation of PCC  It is essential to note that opportunities play a significant role in implementing PCC programs. Factors hindering the implementation of PCC include a lack of coordination in resource allocation and time constraints. Furthermore, strengthening a multidisciplinary team facilitates the development of tailored and comprehensive care plans. Establishing a safe and friendly environment can also facilitate the implementation of PCC. |
| 9.Unfavourable distribution at individual level (remuneration, workload and working conditions) (U) |  |  |
| 12.The orchestrate of resources(U) |  |  |
| 54.Insurance, coverage and benefits(U) |  |  |
| 95.Not be a nuisance to the staff by asking for extra time and help(U) |  |  |
| 98.The service had limited opportunities for involvement and individualized tailored services(C) |  |  |
| 110. Funding was a key limitation to delivering better care(U) |  |  |
| 118. Flexibility of time spent with clients(C) |  |  |
| 122. The importance of resources being available(C) |  |  |
| 8.Lack of coordination and communication regarding clients' care(U) | Strengthening multidisciplinary teamwork |  |
| 18.Organizational teamwork(U) |  |  |
| 39.Enhance feedback with colleagues (U) |  |  |
| 44.Share information with colleagues (C) |  |  |
| 47.Collaboration is easier when older adults and caregivers lead the way(U) |  |  |
| 60.Integrated models of care(U) |  |  |
| 3.On the importance of client’s home environment(U) | Establishing a safe and friendly environment |  |
| 4.On shifting from a providing care expert model to one empowering partnering relationship(U) |  |  |
| 15.Individualized service provision and environmental asp (U) |  |  |
| 26.Engaged participants by forming relationships(U) |  |  |
| 28.Constrained caring relationships(U) |  |  |
| 33.Building a relationship of trust(U) |  |  |
| 45.Building trust and understanding(U) |  |  |
| 48.Older adults and caregivers can direct the care environment(U) |  |  |
| 70.Separate your work environment from your home(U) |  |  |
| 77.Caring relationship(U) |  |  |
| 91. Value on the visits of the homecare providers(U) |  |  |
| 92. the staff were nearby and being safe(U) |  |  |
| 93. The importance of the quality of the relationship with the staff(U) |  |  |
| 94. The desire to live in their own home seemed to increase their effort to adapt to the home care service(U) |  |  |
| 107. Lack of interactions with residents with  stroke-related communication impairment(U) |  |  |
| 111. A homelike atmosphere(C) |  |  |
| 112. A safe environment(C) |  |  |
| 121. A desirable work environment(U) |  |  |
| 17.Limitations to engage in person-centered care (client numbers and lack of a whole service approach) (U) | Time constraints |  |
| 20.On negative impact of staff turnover(U) |  |  |
| 27.Time fostered caring(U) |  |  |
| 31.Little things that went above and beyond(U) |  |  |
| 52.Time to access care(U) |  |  |
| 84.Lack of time(U) |  |  |
| 85.Productivity and compensation(U) |  |  |
| 86.Documentation systems and requirements(U) |  |  |
| 108.Time pressures on care(U) |  |  |
| 109.Staff shortages increased time pressure(U) |  |  |
| 119. Heavy workload and low caseload (C) |  |  |
| 14.Individualized care(U) | Encouraging self-reflection and regulation | Synthesized findings 3: Motivation in the implementation of PCC  Motivation is an important factor influencing behavior change in the implementation of PCC. Encouragement of self-reflection and regulation in practice leads to self-improvement and provide better care services for older people. Respecting the autonomy of older people and maintaining resilient and positive attitudes contribute to the engagement of all stakeholders in the process of PCC. Furthermore, the lack of clear reward and empowerment mechanisms can reduce staff motivation. |
| 16.Set goals that the clients can achieve (U) |  |  |
| 25.Assessing the individual（U） |  |  |
| 30.Respond to differences between individuals(U) |  |  |
| 35.Maintain a critical overview among case managers (U) |  |  |
| 36.Computer-based individual care and support plan(U) |  |  |
| 42.Focused goal-setting(U) |  |  |
| 64.Limited control of patient outcomes(U) |  |  |
| 69.Reflective practice(U) |  |  |
| 80.Creative/critical reflection(U) |  |  |
| 1.Caring as the heart of the practice(U) | Respecting the autonomy of older people |  |
| 2.Focus on the whole aspect based on client-centredness(U) |  |  |
| 10.Being a bit more understanding for older people (U) |  |  |
| 19.Knowing the person（U） |  |  |
| 41.Seeing beyond age enables respect and dignity(U) |  |  |
| 46.Doing ‘with’ instead of doing ‘for’ promotes participation(U) |  |  |
| 51.Availability and appropriateness of care(U) |  |  |
| 53.‘Free’ healthcare(U) |  |  |
| 55.Respectful and compassionate care(U) |  |  |
| 58.Preferences and expectations for care |  |  |
| 59.Equality of care(U) |  |  |
| 61.Informed consent(U) |  |  |
| 62.Patient rights(U) |  |  |
| 63.Approach to Care(U) |  |  |
| 67.Emotional demands(U) |  |  |
| 71.Empathy(U) |  |  |
| 79.Being sensitive to the needs of different people (U) |  |  |
| 87.Business orientation to patient care(U) |  |  |
| 76.Caring values |  |  |
| 102.Lack of sensitivity to the specific needs of older people(U) |  |  |
| 104.Planned activities were not differentiated by ability or inclusive(U) |  |  |
| 105. Respect the autonomy of older people(U) |  |  |
| 106.Residents' interests and preferences were not always supported(U) |  |  |
| 115.Tailoring activities to the clients’ needs and values(U) |  |  |
| 116.Providing them with different levels of assistance by taking into account their disability levels and comorbidities(C) |  |  |
| 5.On encountering disempowering on the system level (governmental ﬁnancial constraint and experienced) (U) | Lack of clear reward and empowerment mechanisms |  |
| 6.On encountering disempowering on the organizational level (centralized allocation and control of service delivery) (U) |  |  |
| 11.Hegemony of the business model of health services Delivery(U) |  |  |
| 13.Delegate authority based on ﬂexible client-driven care(U) |  |  |
| 34.Defining the case manager role(U) |  |  |
| 83.Agency support for patient-centered care(U) |  |  |
| 117. Empowering care providers(C) |  |  |
| 65.Societal views(U) | Having a resilient and optimistic attitude |  |
| 66.Own biases(U) |  |  |
| 68.Acceptance(U) |  |  |
| 73.Congruence(U) |  |  |
| 74.Psychological ﬂexibility(U) |  |  |
| 72.Unconditional positive regard(U) |  |  |
| 88.Side-stepping the barriers with a resilient attitude(U) |  |  |
| 89.Transcending barriers with a resilient attitude(U) |  |  |
