# Supplementary material for: Experiences of older people, healthcare providers and caregivers on implementing person-centered care for community-dwelling older people: a systematic review and qualitative meta-synthesis
Source: BMC Geriatr. 2023 Mar 31;23:207. doi: 10.1186/s12877-023-03915-0 (PMC10067217; doi:10.1186/s12877-023-03915-0)
Supplement: Supplementary file 5 — Additional file 5. [file 12877_2023_3915_MOESM5_ESM.docx]

Additional file 5：ConQual summary of findings

| Systematic review title: Experiences of older people, healthcare providers and caregivers on implementing person-centered care for community-dwelling older people: a systematic review and qualitative meta-synthesis  Population: older people, family caregivers, and healthcare providers  Phenomena of interest: Experiences of older people, healthcare providers and caregivers on person-centered care in community  Context: in community home care | | | | |
| --- | --- | --- | --- | --- |
| Synthesized finding | Type of research | Dependability | Credibility | ConQual score |
| **Capacities of older people, HCPs and caregivers.**  It is crucial to recognize that the capacities of older people, HCPs and caregivers affect the implementation of PCC, including lack of person-centered knowledge and skills, negative attitudes toward shared decision-making and lack of formal training. | Qualitative | Downgrade  1 level* | Downgrade  1 level** | Low |
| **Opportunities in the implementation of PCC**  It is essential to note that opportunities play a significant role in implementing PCC programs. Factors hindering the implementation of PCC include a lack of coordination in resource allocation and time constraints. Furthermore, strengthening a multidisciplinary team facilitates the development of tailored and comprehensive care plans. Establishing a safe and friendly environment can also facilitate the implementation of PCC. | Qualitative | Downgrade  1 level* | Downgrade  1 level** | Low |
| **Synthesized findings 3:** **Motivation in the implementation of PCC**  Motivation is an important factor influencing behavior change in the implementation of PCC. Encouragement of self-reflection and regulation in practice leads to self-improvement and provide better care services for older people. Respecting the autonomy of older people and maintaining resilient and positive attitudes contribute to the engagement of all stakeholders in the process of PCC. Furthermore, the lack of clear reward and empowerment mechanisms can reduce staff motivation. | Qualitative | Downgrade  1 level* | Downgrade  1 level** | Low |

*Downgraded one level due to common dependability issues across the included primary studies (the majority of studies did not present a statement locating the researcher culturally or theoretically, and there was no acknowledgment of their influence on the research).

**Downgraded one level to a mix of unequivocal and credible findings.
