# Supplementary material for: Experiences of older people, healthcare providers and caregivers on implementing person-centered care for community-dwelling older people: a systematic review and qualitative meta-synthesis
Source: BMC Geriatr. 2023 Mar 31;23:207. doi: 10.1186/s12877-023-03915-0 (PMC10067217; doi:10.1186/s12877-023-03915-0)
Supplement: Supplementary file 6 — Additional file 6. [file 12877_2023_3915_MOESM6_ESM.docx]

Additional file 6: Recommendation for practice

| Recommendation | Joanna Briggs Institute Grade |
| --- | --- |
| The community managers should provide professional training to improve person-centered knowledge and skills for older people, HCPs and caregivers. | A |
| We should encourage HCPs to engage community-dwelling older people in shared decision-making about self-management goals and actions. | A |
| Community managers should conduct rational coordination of resource allocation to support changes at the individual and environmental levels by providing manpower arrangements and suitable working conditions. | B |
| We should establish and integrate the multidisciplinary team to implement effective PCC programs in the community. | A |
| We should establish a desirable environment for older people and HCPs, especially a trustful relationship between them. | A |
| All stakeholders should realize that time constraints and limited HCPs make it challenging to implement effective PCC. Community institutions should clarify each employee's work responsibilities and allocate time reasonably. | A |
| The HCPs and caregivers should consider compassionate caring as the heart of the practice and respect the autonomy of older people. | A |
| Community managers should establish clear rewards and accountability mechanisms to strengthen the involvement of HCPs and professionalize management. | A |
| The HCPs should keep being resilient and optimistic and learn to adjust their roles in the face of setbacks. | B |
